# Cytotoxic, Antitumor and Immunomodulatory Effects of the Water-Soluble Polysaccharides from Lotus (*Nelumbo nucifera* Gaertn.) Seeds

**DOI:** 10.3390/molecules21111465

**Published:** 2016-11-02

**Authors:** Yafeng Zheng, Qi Wang, Weijing Zhuang, Xu Lu, Anca Miron, Tsun-Thai Chai, Baodong Zheng, Jianbo Xiao

**Affiliations:** 1 College of Food Science, Fujian Agriculture and Forestry University, Fuzhou 350002, China; zyffst@163.com (Y.Z.); nkywq@163.com (Q.W.); newfourtharmy@163.com (W.Z.); luxuluxu88@163.com (X.L.); 2 Institute of Agricultural Engineering, Fujian Academy of Agriculture Sciences, Fuzhou 350003, China; 3 Faculty of Pharmacy, Grigore T. Popa University of Medicine and Pharmacy Iasi, Universitatii Street, No. 16, Iasi 700115, Romania; ancamiron@yahoo.com or anca.miron@umfiasi.ro; 4 Department of Chemical Science, Faculty of Science, Universiti Tunku Abdul Rahman, Jalan Universiti Bandar Barat, Kampar 31900, Perak, Malasia; chaitt@utar.edu.my; 5 Institute of Chinese Medical Sciences, State Key Laboratory of Quality Research in Chinese Medicine, University of Macau, Taipa, Macau, China

**Keywords:** lotus seeds, polysaccharides, antitumor, immunomodulatory

## Abstract

Lotus is an edible and medicinal plant, and the extracts from its different parts exhibit various bioactivities. In the present study, the hot water–soluble polysaccharides from lotus seeds (LSPS) were evaluated for their cancer cell cytotoxicity, immunomodulatory and antitumor activities. LSPS showed significant inhibitory effects on the mouse gastric cancer MFC cells, human liver cancer HuH-7 cells and mouse hepatocarcinoma H22 cells. The animal studies showed that LSPS inhibited tumor growth in H22 tumor-bearing mice with the highest inhibition rate of 45.36%, which is comparable to that induced by cyclophosphamide (30 mg/kg) treatment (50.79%). The concentrations of white blood cells were significantly reduced in cyclophosphamide-treated groups (*p* < 0.01), while LSPS showed much fewer side effects according to the hematology analysis. LSPS improved the immune response in H22 tumor-bearing mice by enhancing the spleen and thymus indexes, and increasing the levels of serum cytokines including tumor necrosis factor-α and interleukin-2. Moreover, LSPS also showed in vivo antioxidant activity by increasing superoxide dismutase activity, thus reducing the malondialdehyde level in the liver tissue. These results suggested that LSPS can be used as an antitumor and immunomodulatory agent.

## 1. Introduction

Cancer is a malignant disease characterized by an abnormal cell growth and proliferation with the potential to invade surrounding tissues and metastasize to other tissues in the body [1]. As cancer incidence increases every year, more effective therapies are urgently needed. However, currently many available chemotherapy drugs cause serious side effects [2]. Previous studies showed that different non-toxic polysaccharides extracted from natural sources exhibit promising antitumor and immunomodulatory activities [3].

**Figure 1:**
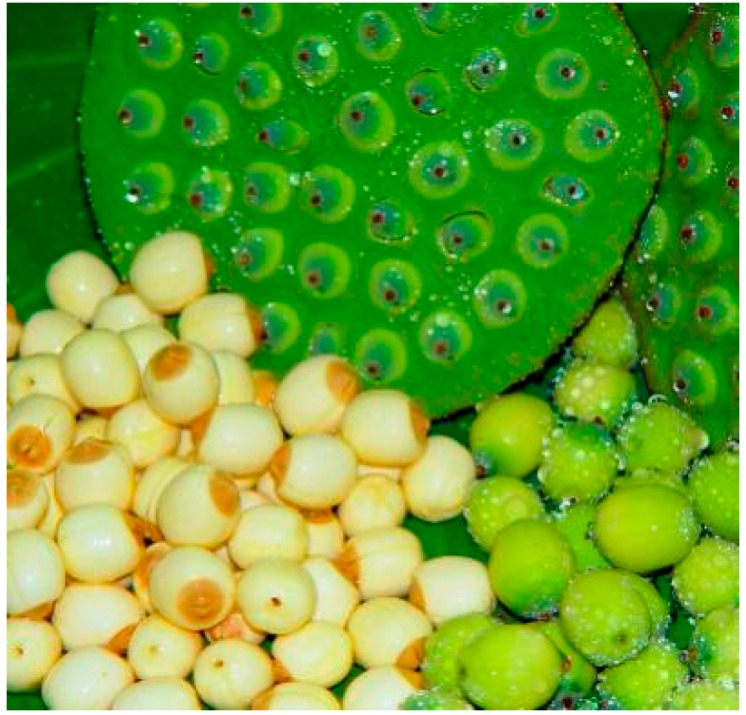
Seeds and seedpods of lotus (*Nelumbo nucifera* Gaertn.).

Lotus (*Nelumbo nucifera* Gaertn.), an aquatic perennial plant of economic importance, has been cultivated in China for about 3000 years. Being both an edible and medicinal plant, all the parts of the lotus are used, especially the rhizomes and seeds [4]. Lotus seeds (Figure 1), called ‘Lian Zi’ in Chinese, are valuable sources of proteins, starch, and unsaturated fatty acids, which have a well-balanced amino acid composition, as well as low levels of sugar, sodium and cholesterol [5,6]. Besides being used as food, lotus seeds also have a very long history as a traditional medicine in China and are used as diuretic, antiemetic, anti-inflammatory and anticancer agents [7,8]. Various active components extracted from different part of lotus seeds (seedpod, plumule and cotyledons) were characterized to be mainly flavonoids, polyphenols and alkaloids [9], which have many pharmacological and physiological activities, including antioxidant activity [10,11,12,13], DNA- and cell-protective effects [14,15], anti-obesity activity [16], immunomodulatory effects [17], and anti-cancer activity [18,19].

Isolation and characterization of polysaccharides with antitumor activity, an immunomodulatory effect or other pharmacology have emerged as one of the important research fields in chemistry and biology [20]. However, there is little data on the lotus polysaccharides. Jiang and coworkers reported that a polysaccharide-protein complex from the lotus rhizome exhibited a certain immunoregulatory effect and anti-HIV-1 activity [21]. Moreover, the lotus plumule polysaccharide was purified, characterized and proved to exert anti-inflammatory activity and anti-diabetic activity [22,23,24,25,26]. In our previous study, a novel water-soluble polysaccharide from lotus seed was extracted, characterized and found to exhibit antioxidant potential [27]. Further investigation is required to reveal the potency of this novel lotus seed polysaccharide. Therefore, the present study was carried out to evaluate the cytotoxic, antitumor and immunomodulatory effects of the hot water–soluble polysaccharides from lotus seeds.

## 2. Results

### 2.1. Extraction of Lotus Seed Polysaccharides

LSPS were extracted from the dried lotus seeds by hot water extraction. The extraction yield of LSPS was 0.95%, which is lower than the yield (1.51%) of the lotus seeds’ polysaccharides using ultrasound-assisted extraction as reported in our previous study [27]. However, lotus seeds are a traditional food ingredient in various soups. The polysaccharides obtained by hot water extraction are more similar to the dietary ones present in lotus seed soups. A comparative analysis of the structural properties of the lotus seed polysaccharides extracted by various methods will be performed in our future studies.

### 2.2. Cytotoxic Effects on MFC, HuH-7 and H22 Cells

**Figure 2:**
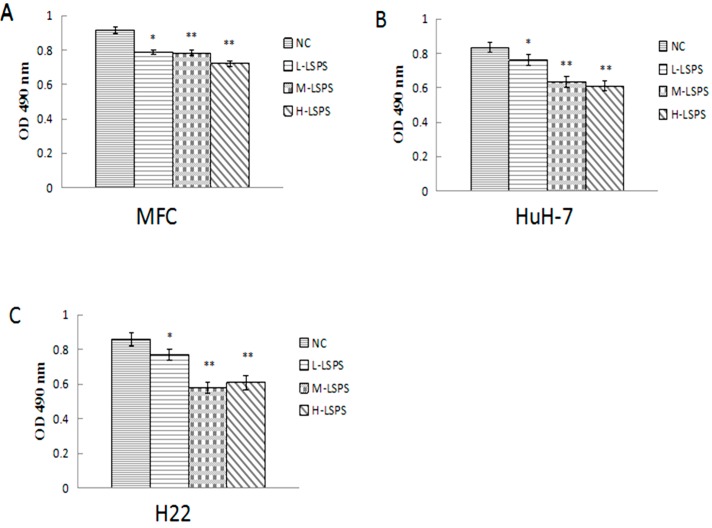
Inhibitory effect of LSPS on MFC (**A**); HuH-7 (**B**) and H22 (**C**) cells. The OD values are represented as means ± SD. Compared with negative control (NC), * *p* < 0.05; ** *p* < 0.01. L-LSPS, incubated with lotus seed polysaccharide solution (50 μg/mL); M-LSPS, incubated with lotus seed polysaccharide solution (100 μg/mL); H-LSPS, incubated with lotus seed polysaccharide solution (200 μg/mL).

To evaluate the cytotoxicity of LSPS on tumor cells in vitro, MFC, HuH-7 and H22 cells were incubated with various concentrations of LSPS (0, 50, 100 and 200 μg/mL) for 48 h. According to Figure 2, compared with the NC group, incubation of various concentrations of LSPS inhibited the growth of tumor cells. However, all the doses of LSPS in tumor cell culture media are not sufficient for half-maximal inhibition of tumor cell proliferation, suggesting that LSPS could only weakly inhibit tumor cell proliferation in vitro [28]. It is noticeable that LSPS exhibited more potent inhibitory effects on viability in HuH-7 and H22 cells in comparison to MFC cells, which suggests a certain antitumor potential, especially on hepatoma cells.

### 2.3. Effect of LSPS on Tumor Growth and Body Weight

**Figure 3:**
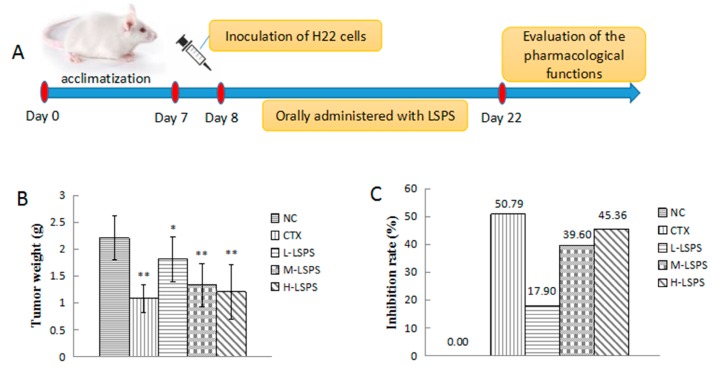

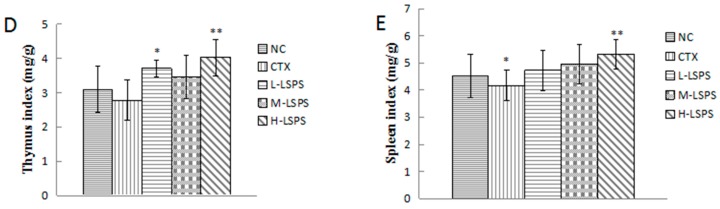
In vivo antitumor activities of LSPS in H22 tumor-bearing mice. Experiment design of H22 mouse hepatocellular carcinoma model (**A**); Effect of LSPS on tumor weight (**B**); and average tumor inhibition rate (**C**); Effect of LSPS on thymus index (**D**); and spleen index (**E**). The results are expressed as means ± SD of 10 separate experiments. Compared with negative control (NC), * *p* < 0.05; ** *p* < 0.01. CTX, cyclophosphamide (30 mg/kg) treated group; L-LSPS, lotus seed polysaccharide (50 mg/kg) treated group; M-LSPS, lotus seed polysaccharide (100 mg/kg) treated group; H-LSPS, lotus seed polysaccharide (200 mg/kg) treated group.

To assess the antitumor activity of LSPS in vivo, LSPS was orally administered to H22 tumor-bearing mice for 14 days. A significant reduction in the tumor weight was detected in the treated groups at the end of the experimental period, and the results are summarized in Figure 3. Compared to the negative control group, the CTX-treated group showed significant tumor inhibition (50.79%, *p* < 0.01). Meanwhile, the different doses of LSPS (50, 100 and 200 mg/kg) resulted in tumor inhibition in H22 tumor-bearing mice with rates of 17.90%, 39.60% and 45.36%, respectively. 

**Figure 4:**
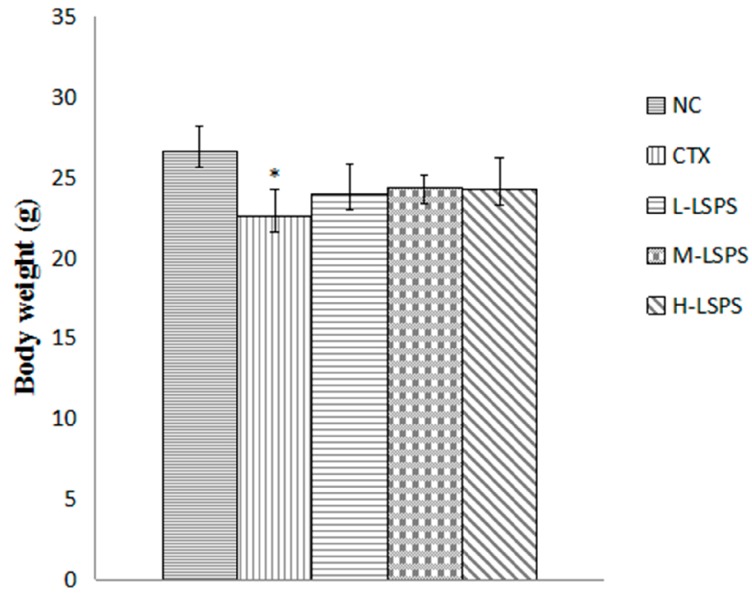
Effects of LSPS on body weight in H22 tumor-bearing mice. The results are expressed as means ± SD of 10 separate experiments. Compared with negative control (NC), * *p* < 0.05. CTX, cyclophosphamide (30 mg/kg) treated group; L-LSPS, lotus seed polysaccharide (50 mg/kg) treated group; M-LSPS, lotus seed polysaccharide (100 mg/kg) treated group; H-LSPS, lotus seed polysaccharide (200 mg/kg) treated group.

Furthermore, due to the relatively low toxicity, the appetite, activity and coat luster of each animal in LSPS-treated groups were better than those of CTX-treated mice. Moreover, the body weight variations in each group also indicated a low toxicity of LSPS (Figure 4). Before the treatment, the body weights in each group were similar. After the 14-day treatment, due to CTX toxicity, the body weight in the CTX-treated group showed a significantly decreasing trend (*p* < 0.05) in comparison to that of the control group, while the body weight in the LSPS-treated groups showed no significant change.

### 2.4. Effect of LSPS on Immune Organs

According to previous studies, the antitumor activity of polysaccharides is usually related to the stimulation of the immune response [29,30,31]. The in vivo immunomodulatory effect of LSPS was evaluated by the immune organ (thymus and spleen) indexes in H22 tumor-bearing mice (Figure 3). Compared to the negative control, the CTX treatment caused a 7.92% and 10.19% reduction in the spleen and thymus indexes, respectively, indicating that CTX suppresses the immunological function in mice. However, LSPS administration obviously increased the spleen and thymus indexes, compared to the negative control group. The effect of LSPS on immune organs did not strictly follow the dose-dependency trend, and the low dose of LSPS showed a more pronounced effect on the thymus index than the medium dose. These results suggest that the antitumor activity of LSPS might be related to the activation of the immune responses in the host, whereas its side effects are lower than those induced by CTX [32].

### 2.5. Effect of LSPS on Blood Physiochemical Parameters

**Figure 5:**
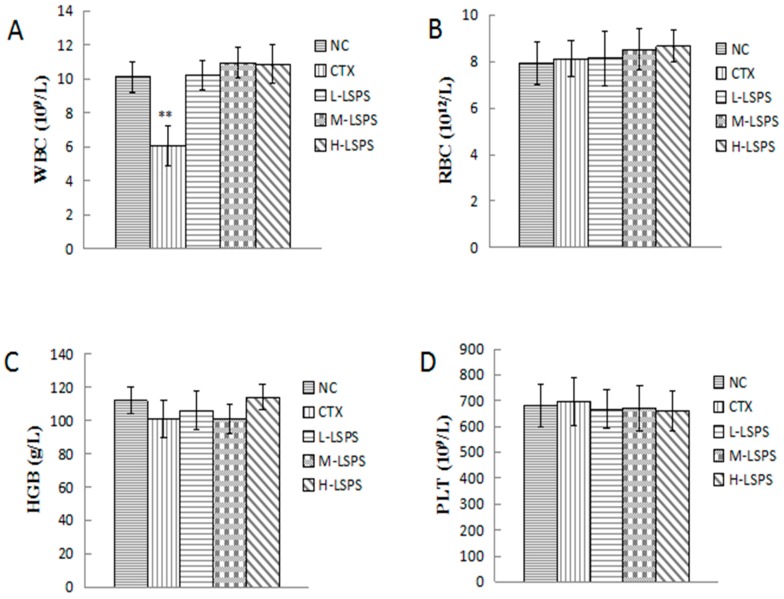
Effects of LSPS on WBC (**A**); RBC (**B**); HGB (**C**); and PLT (**D**) in H22 tumor-bearing mice. The results are expressed as means ± SD of 10 separate experiments. Compared with negative control (NC), ** *p* < 0.01. WBC, white blood cells; RBC, red blood cells, HGB, hemoglobin; PLD, platelets; CTX, cyclophosphamide (30 mg/kg) treated group; L-LSPS, lotus seed polysaccharide (50 mg/kg) treated group; M-LSPS, lotus seed polysaccharide (100 mg/kg) treated group; H-LSPS, lotus seed polysaccharide (200 mg/kg) treated group.

As shown in Figure 5, compared with that in the NC group (administrated with saline), the CTX injection was found to dramatically decrease the number of white blood cells (*p* < 0.01), indicating a strong toxic side effect on the immune system of the host. In contrast, the concentrations of white blood cells (WBC) in all the LSPS-treated groups were not significantly changed. The other blood parameters, including red blood cells (RBC), hemoglobin (HGB) and platelets (PLD), were found to show no significant changes after CTX or LSPS administration. These results suggested that, compared with CTX, LSPS caused fewer side effects or toxicity to the host. 

### 2.6. Effect of LSPS on Serum TNF-α and IL-2 Levels

**Figure 6:**
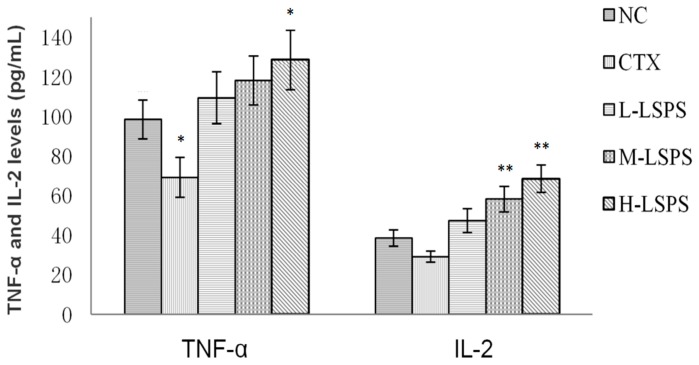
Effects of LSPS on serum TNF-α and IL-2 levels in H22 tumor-bearing mice. The results are expressed as means ± SD of 10 separate experiments. Compared with negative control (NC), * *p* < 0.05; ** *p* < 0.01. CTX, cyclophosphamide (30 mg/kg) treated group; L-LSPS, lotus seed polysaccharide (50 mg/kg) treated group; M-LSPS, lotus seed polysaccharide (100 mg/kg) treated group; H-LSPS, lotus seed polysaccharide (200 mg/kg) treated group.

The effects of LSPS on TNF-α and IL-2 levels in the serum of H22 tumor-bearing mice were investigated (Figure 6). The serum TNF-α and IL-2 levels decreased in CTX-treated mice compared to the negative control, most probably due to the immunosuppressive effects of CTX. However, in the LSPS-treated groups, the production of TNF-α and IL-2 was enhanced, indicating that LSPS administration could improve immune function in H22 tumor-bearing mice by promoting TNF-α and IL-2 production. 

### 2.7. Effect of LSPS on SOD Activity and MDA Level 

According to the results shown in Table 1, unlike the negative control group, the administration of different doses (50, 100 and 200 mg/kg) of LSPS increased SOD activity in the liver tissue by 5.5%, 14.4% and 15.2%, respectively. Moreover, the levels of MDA in the liver tissue in the low-, medium- and high-dose LSPS-treated groups were reduced by 8.6%, 12.9% and 20.5%, respectively. However, the administration of CTX suppressed SOD activity in the liver, while the MDA level was still lower than that of the negative control group. These results suggest that LSPS improved SOD activity, thus leading to a decrease in oxidative DNA and tissue damage; both effects (the increase in SOD activity and the decrease in MDA level) might contribute to the antitumor activity of LSPS.

## 3. Discussion

According to the reports of the World Health Organization, the number of cancer cases around the world is expanding rapidly and is expected to reach 24 million by 2035 [33]. Although many antitumor drugs exhibit promising therapeutic effects, their use is still greatly hampered by a high toxicity to normal cells, both the body health and life quality being significantly affected [34]. In search for novel antitumor agents with much less toxicity, polysaccharides from various natural sources are becoming more and more attractive in current scenarios due to their safety for human health and their immune-based antitumor activity (activation of the host immune defense mechanisms with a consequent indirect inhibition of the tumor cell growth) [3,31,35].

Lotus seeds have been used as a top tonic since ancient in China. Recent studies have shown that lotus seeds exhibit various bioactivities, including gastrointestinal regulation, and antioxidant, hypoglycemic and immunomodulatory effects [4]. In our previous study, ultrasound-assisted extraction was used to extract a novel lotus seed polysaccharide that was successively purified to yield a major low-molecular-weight polysaccharide fraction (4.5 kDa), which exhibited a potent antioxidant activity in a dose-dependent manner [27].

The antitumor and immunomodulatory effects of LSPS were investigated in this study. CTX is an alkylating agent commonly used in antitumor therapy, but also in the immunosuppressive therapy [36]. Thus, a CTX-treated group was used as a positive control. Compared to the negative control group treated with saline, LSPS administration showed a weak inhibitory effect on the tumor cells, including the mouse gastric cancer MFC cells, human liver cancer HuH-7 cells and mouse hepatocarcinoma H22 cells. In the previous antitumor studies, some polysaccharides were reported to exhibit strong cytotoxic effects on tumor cells [37,38,39], while some other antitumor polysaccharides showed fewer or no effects on tumor cell proliferation in vitro [40]. Oral administration of LSPS showed a promising antitumor activity comparable to that of CTX in H22 tumor-bearing mice. As a natural antitumor defense system, the immune system plays an important role in the response to antitumor therapy. The antitumor effect of LSPS could be attributed to its positive influence on the immune organ index, serum cytokines and antioxidant enzymes.

The variation trend of spleen and thymus indexes reflects the immune function of the organism [41]. CTX treatment suppressed the tumor growth, but significantly decreased both spleen and thymus indexes. On the contrary, LSPS administration increased the weight of the spleen and thymus, indicating immunopotentiating effects. Cytokines are small proteins produced by cells that exert important effects on cell signaling. They play an important role in host responses to infection, inflammation, and cancer, but also in reproduction [42]. TNF-α and IL-2 are cytokines possessing antitumor and immunomodulatory properties. According to the data obtained for serum samples of LSPS-treated mice, TNF-α and IL-2 levels were enhanced by LSPS treatment. These results were consistent with those from other studies reporting the ability of polysaccharides to activate the immune system partially by enhancing TNF-α and IL-2 production [43,44,45]. SOD is a primary antioxidant enzyme in mammals; it protects the tissues against excessive oxidative damage, which might lead to DNA lesions and finally carcinogenesis. MDA is a final product of lipid peroxidation, and has been used as a biological marker for oxidative stress. The antitumor effects of LSPS could be partially related to its antioxidant activity (increase in SOD activity, decrease in MDA level) in the liver tissue [44].

In this study, the results suggested that a high dose is needed for LSPS efficacy. However, the extraction yield of LSPS in hot water was only 0.95%, indicating that the dietary intake of lotus seed polysaccharides is not sufficient for tumor prevention and therapy. Therefore, the development of a LSPS-enriched food additive or functional food is needed.

## 4. Materials and Methods

### 4.1. Materials and Reagents

The lotus seed Jianxuan No. 17, a popular cultivar in Jianning County (Fujian, China), was purchased from a local market and identified by Jingdong Wu, senior agronomist of The Lotus Research Institute of Jianning County. The lotus seeds were ground with a mill and sieved, then sealed in air-tight plastic bags for further application. Cyclophosphamide was purchased from Jiangsu Hengrui Co. (Lianyungang, China). The 3-(4,5-Dimethylthiazol-2-yl)-2,5-diphenyltetrazolium bromide (MTT) was provided by Sigma Chemical Co. (St. Louis, MO, USA). RPMI 1640 medium and fetal bovine serum (FBS) were obtained from Gibco Life Technologies (Carlsbad, CA, USA). Dimethyl sulfoxide (DMSO), TNF-α and interleukin-2 detecting ELISA kits were purchased from Boster Bioengineering Co. (Wuhan, China). All other reagents were of analytical grade.

### 4.2. Extraction of the Hot Water–Soluble Polysaccharides

The lotus seed powder was extracted with 10-fold volume of hot water (100 °C) for 3 h [35]. After extraction, the supernatant and sediment were separated by vacuum filtration. The extractive solution was concentrated using a rotary evaporator at 55 °C under vacuum. Then four-fold volume of ethanol (95%, *v*/*v*) was added and stored overnight at 4 °C. The precipitate was solubilized in deionized water and deproteinized by plumbous acetate (10%, *w*/*w*). After centrifugation, the supernatant was concentrated and dialyzed for 48 h at 4 °C and was further vacuum freeze-dried to yield the crude lotus seeds polysaccharides (LSPS).

### 4.3. Cell Lines and Animals

Mouse gastric cancer MFC cells, human liver cancer HuH-7 cells and mouse hepatocarcinoma H22 cells were offered by Fujian Medical University (Fuzhou, China). These cells were grown in RPMI 1640 medium supplemented with 10% FBS, 100 U/mL penicillin and streptomycin at 37 °C with 5% CO_2_.

Kunming mice (female, grade II, six to eight weeks old, 20.0 ± 2.0 g, SCXK (hu) 2012-0002) were purchased from SLAC Laboratory Animal Co. (Shanghai, China). The mice were housed under room temperature (23 ± 2 °C), humidity of 50% ± 10%, 12/12 h light-dark cycle with free access to standard laboratory pellet diet and water during the experiments. All the experimental procedures were in accordance with EU Directive 2010/63/EU for the protection of animals used for scientific purposes, and approved by the Animal Care Review Committee, Fujian Agriculture and Forest University, China.

### 4.4. In Vitro Cytotoxicity on MFC, HuH-7 and H22 Cell Lines

In vitro cytotoxicity of LSPS on MFC, HuH-7 and H22 cell lines was evaluated by MTT assay, as described by Thetsrimuang and coworkers with minor modifications [37]. The cells were washed and maintained in RPMI 1640 medium (containing 10% FBS) at a concentration of 5 × 10^5^ cells/mL in 96-well plates. After 24 h incubation at 37 °C, LSPS solution (0, 50, 100 and 200 μg/mL) was added to the cells, and incubated for 48 h. After incubation, the supernatant was removed and 100 μL of MTT solution (1 mg/mL) was added to each well. The plate was further incubated at 37 °C for 4 h. The medium was removed by centrifugation, and 100 μL of DMSO was added to each well. Finally, the absorbance at 490 nm was determined using a Bio-Rad micro-plate reader (Hercules, CA, USA). The growth inhibition rate (%) of tumor cells was calculated as [(A − B)/A × 100], where A and B are the absorbance of the control and LSPS-treated group, respectively. All tests were repeated in triplicate.

### 4.5. In Vivo Antitumor Activity in H22 Mice Hepatocellular Carcinoma Model

To elucidate the antitumor activity of LSPS in vivo, H22 mice hepatocellular carcinoma model was applied on Kunming mice after one week acclimatization. H22 cell suspension (0.2 mL, 1 × 10^7^ cells/mL) was inoculated into the axilla of the left foreleg of Kunming mice under sterile condition. After 24 h, the mice were randomly divided into five groups (10 mice in each group), namely negative control group (NC), CTX-treated group (CTX), and three LSPS-treated groups. The mice in the LSPS-treated groups were orally administered with LSPS in saline at low (L, 50 mg/kg), medium (M, 100 mg/kg) and high (H, 200 mg/kg) doses once daily consecutively for 14 days. The CTX-treated mice were intraperitoneally injected with CTX (30 mg/kg) for 14 days, whereas the negative control mice received the same volume of saline.

On the next day after the last administration, the mice were weighed and sacrificed by cervical dislocation. The tumors were carefully dissected and weighed, and the antitumor activity of LSPS was expressed as tumor inhibition percentage (%). The thymus and spleen were dissected and weighed. The effect on the spleen weight was expressed as the spleen index calculated as spleen (mg)/body weight (g). The thymus index was calculated using the same procedure. The blood samples were collected from the mice’s eyes, and the serum samples were collected by centrifugation at 3000 rpm at 4 °C for 10 min.

### 4.6. Determination of Blood Physiochemical Parameters

Hematology analysis was carried out to verify the effects of LSPS administration on H22 tumor-bearing mice. Blood sample (20 μL) was collected from each mouse at the end of experiment, and the concentrations of white blood cells (WBC), red blood cells (RBC), hemoglobin (HGB), and platelets (PLD) were determined by an automatic blood cell counting apparatus (Mindray, Shenzhen, China) according to the instructions.

### 4.7. Determination of Serum TNF-α and IL-2 Levels

The collected serum samples were used for the determination of TNF-α and IL-2 levels using commercial ELISA kits. The absorbance was measured at 450 nm using an ELISA reader (Bio-Rad).

### 4.8. Determination of SOD Activity and MDA Level

Mice livers were carefully taken and homogenized in nine volumes of ice-cold 0.9% saline solution to prepare 10% (*w*/*v*) liver homogenates, which were further centrifuged at 3500 rpm at 4 °C for 10 min. SOD activity and MDA levels in the homogenates were assessed according to the kit instructions.

### 4.9. Statistical Analysis

Data were analyzed using SPSS software (11.5, SPSS, Inc., Chicago, IL, USA), and the results were expressed as means ±SD. Data in all the bioassays were statistically evaluated by analysis of variance and *p*-values of 0.05 or less were considered statistically significant.

## 5. Conclusions 

LSPS was isolated with a yield of 0.95% from dried lotus seed powder by hot water extraction. LSPS showed inhibitory effects on the mouse gastric cancer MFC cells, human liver cancer HuH-7 cells and mouse hepatocarcinoma H22 cells. In H22 tumor-bearing mice, LSPS inhibited the tumor growth with the highest inhibition rate of 45.36%, which is comparable to that induced by CTX (30 mg/kg) treatment (50.79%). Based on the results of the hematology analysis, LSPS was proved to have much fewer side effects than that of CTX, which significantly reduced the concentrations of white blood cells in CTX-treated groups (*p* < 0.01). The antitumor activity of LSPS mainly relies on its effects on the immune organ index, serum cytokines and antioxidant enzymes. LSPS administration increased both the spleen and thymus indexes, and the serum levels of TNF-α and IL-2. Moreover, LSPS also increased SOD activity, and thus reduced the MDA content in the liver tissue. These results suggested that LSPS has potential for use as an antitumor and immunomodulatory agent; however, the detailed mechanism underlying its antitumor activity and immunomodulatory effect should be further evaluated through additional methods and techniques in our further research.

## Figures and Tables

**Table 1:** Effect of LSPS on SOD activity and MDA level in the liver of H22 tumor-bearing mice.

	NC	CTX	L-LSPS	M-LSPS	H-LSPS
SOD (U/mg protein)	140.09 ± 10.16	130.69 ± 11.23	147.81 ± 9.68	160.29 ± 13.89 *	161.39 ± 13.18 *
MDA (nmol/mg protein)	2.10 ± 0.19	2.01 ± 0.24	1.92 ± 0.19	1.83 ± 0.24 *	1.67 ± 0.21 **

The results are expressed as means ± SD of 10 separate experiments. Compared with negative control (NC), * *p* < 0.05, ** *p* < 0.01. CTX, cyclophosphamide (30 mg/kg) treated group; L-LSPS, lotus seed polysaccharide (50 mg/kg) treated group; M-LSPS, lotus seed polysaccharide (100 mg/kg) treated group; H-LSPS, lotus seed polysaccharide (200 mg/kg) treated group.
